# Radiocobalt-Labeling of a Polypyridylamine Chelate Conjugated to GE11 for EGFR-Targeted Theranostics

**DOI:** 10.3390/molecules30020212

**Published:** 2025-01-07

**Authors:** Lorraine Gaenaelle Gé, Mathias Bogetoft Danielsen, Aaraby Yoheswaran Nielsen, Mathias Lander Skavenborg, Niels Langkjær, Helge Thisgaard, Christine J. McKenzie

**Affiliations:** 1 Department of Nuclear Medicine, Odense University Hospital, Kloevervaenget 47, 5000 Odense C, Denmark; lorrainege94@hotmail.com (L.G.G.); aaraby.yoheswaran.nielsen@rsyd.dk (A.Y.N.); niels.langkjaer@rsyd.dk (N.L.); 2 Department of Clinical Research, University of Southern Denmark, Campusvej 55, 5230 Odense M, Denmark; 3 Department of Physics, Chemistry and Pharmacy, University of Southern Denmark, Campusvej 55, 5230 Odense M, Denmark; mbd@sdu.dk (M.B.D.); skavenborg@sdu.dk (M.L.S.)

**Keywords:** theranostics, Co isotopes (^58m^Co, ^57^Co and ^55^Co), polypyridyl chelator, GE11, EGFR targeting

## Abstract

The overexpression of the epidermal growth factor receptor (EGFR) in certain types of prostate cancers and glioblastoma makes it a promising target for targeted radioligand therapy. In this context, pairing an EGFR-targeting peptide with the emerging theranostic pair comprising the Auger electron emitter cobalt-58m (^58m^Co) and the Positron Emission Tomography-isotope cobalt-55 (^55^Co) would be of great interest for creating novel radiopharmaceuticals for prostate cancer and glioblastoma theranostics. In this study, GE11 (YHWYGYTPQNVI) was investigated for its EGFR-targeting potential when conjugated using click chemistry to N1-((triazol-4-yl)methyl)-N1,N2,N2-tris(pyridin-2-ylmethyl)ethane-1,2-diamine (TZTPEN). This chelator is suitable for binding Co^2+^ and Co^3+^. With cobalt-57 (^57^Co) serving as a surrogate radionuclide for ^55/58m^Co, the novel GE11-TZTPEN construct was successfully radiolabeled with a high radiochemical yield (99%) and purity (>99%). [^57^Co]Co-TZTPEN-GE11 showed high stability in PBS (pH 5) and specific uptake in EGFR-positive cell lines. Disappointingly, no tumor uptake was observed in EGFR-positive tumor-bearing mice, with most activity being accumulated predominantly in the liver, gall bladder, kidneys, and spleen. Some bone uptake was also observed, suggesting in vivo dissociation of ^57^Co from the complex. In conclusion, [^57^Co]Co-TZTPEN-GE11 shows poor pharmacokinetics in a mouse model and is, therefore, not deemed suitable as a targeting radiopharmaceutical for EGFR.

## 1. Introduction

The Auger electron emitter (AEE) cobalt-58m (^58m^Co) and the Positron Emission Tomography (PET)-isotope cobalt-55 (^55^Co) form a true theranostic pair, with particularly suitable properties for therapy and diagnostics: ^55^Co emits positrons (76%) with an average energy of 570 keV and has a half-life of 17.5 h, making it an excellent PET-isotope [1,2,3,4,5,6,7,8,9,10]. The promising therapeutic properties of the AEE, ^58m^Co (T_1/2_ = 9.1 h), have been theoretically predicted and confirmed by in vitro and in vivo experiments using PSMA and octreotide conjugates of DOTA [2,11,12]. In our efforts toward the clinical implementation of cobalt radionuclides, we are investigating polydentate polypyridyl-amine chelators, which had not previously been applied in radiochemistry. This is even though this class of chelator has been shown to bind first-row metal ions with very high stability constants [13], in some cases exceeding those of ethylenediaminetetraacetic acid (EDTA) for the same metal ions [14]. N1,N1,N2,N2-tetrakis(pyridin-2-ylmethyl)ethane-1,2-diamine (TPEN) is an example of a hexadentate polypyridyl-amine chelator. The stability constant for [Co^II^(TPEN)]^2+^ (Log(β) = 16.6) [13] shows the same order of magnitude as the binding constant for [Co^II^(EDTA)]^2-^ (Log(β) = 16.1) [14] under comparable conditions.

To optimize treatment, radionuclide vectors must specifically target cancer cells. Overexpression of the epidermal growth factor receptor (EGFR) in primary tumors of prostate cancer and in high-grade gliomas, such as glioblastoma, often correlates with a poorer prognosis [15,16,17,18,19,20,21,22]. Thus, radiolabeled peptides that bind to EGFR for tumor imaging and therapy are of great interest. We, therefore, aimed for the conjugation of a polypyridyl-amine chelator to an EGFR-targeting peptide. Li et al. have identified GE11 (letter code: YHWYGYTPQNVI) to be a potential EGFR-targeting peptide by screening a phage display peptide library [23]. GE11 is proposed to bind to the same binding pocket as one of the natural EGFR ligands, epidermal growth factor (EGF) [23,24]. In contrast to EGF, GE11 has been shown to exhibit a low mitogenic effect, thus limiting the proliferation of tumor cells [23,25]. Therefore, for our initial foray into this chemical space, we chose to deploy GE11 to develop a synthetic protocol for peptide conjugation with a polypyridyl-amine chelator. Conflicting results pointing both to specific targeting [23,25,26,27,28,29,30,31,32,33] and to little or no specific binding in vitro and in vivo have, however, emerged from investigations of GE11 attached to radiolabels [24,34,35,36]. To what extent these discrepancies might be due to variations in the chelator, radioisotope, linker, and number of GE11 peptides in the different radiopharmaceutical constructs is unclear. All are factors that influence lipophilicity, charge, stability, and molecular size [37,38,39,40,41,42,43,44].

**Figure 1:**
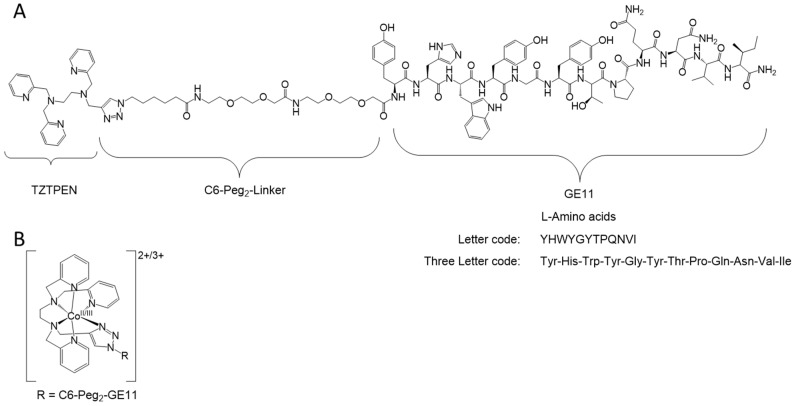
(**A**) The chemical structure of TZTPEN-GE11 containing an amidated C-termini. (**B**) Proposed structure of the labeled end of Co(II/III)-TZTPEN-GE11.

The novel TZTPEN-GE11 construct (Figure 1A) comprises two consecutive polyethylene glycol (PEG) spacers linking the GE11 to the triazole group of N1-((triazol-4-yl)methyl)-N1,N2,N2-tris(pyridin-2-ylmethyl)ethane-1,2-diamine (TZTPEN), with the proposed coordination depicted in Figure 1B. Using ^57^Co as a surrogate for ^58m^Co and ^55^Co, the aim was to test the affinity of TZTPEN-GE11 for radioactive Co under typical labeling conditions and perform a pre-clinical evaluation of the [^57^Co]-labeled system to determine its suitability for PET imaging and therapy of EGFR-positive prostate cancer and glioblastoma. ^58m^Co and ^55^Co are both readily produced in high yields using a small biomedical cyclotron (e.g., a GE PETtrace), as demonstrated previously [1−5,10,11]. Satisfyingly, TZTPEN-GE11 is readily labeled by ^57^Co, with exceptionally high yields; however, despite some EGFR-specific cell uptake, mice studies indicate that it is not suitable for further biological investigations using radioactive cobalt.

## 2. Results and Discussion

### 2.1. TZTPEN-GE11 Synthesis

**Scheme 1:**
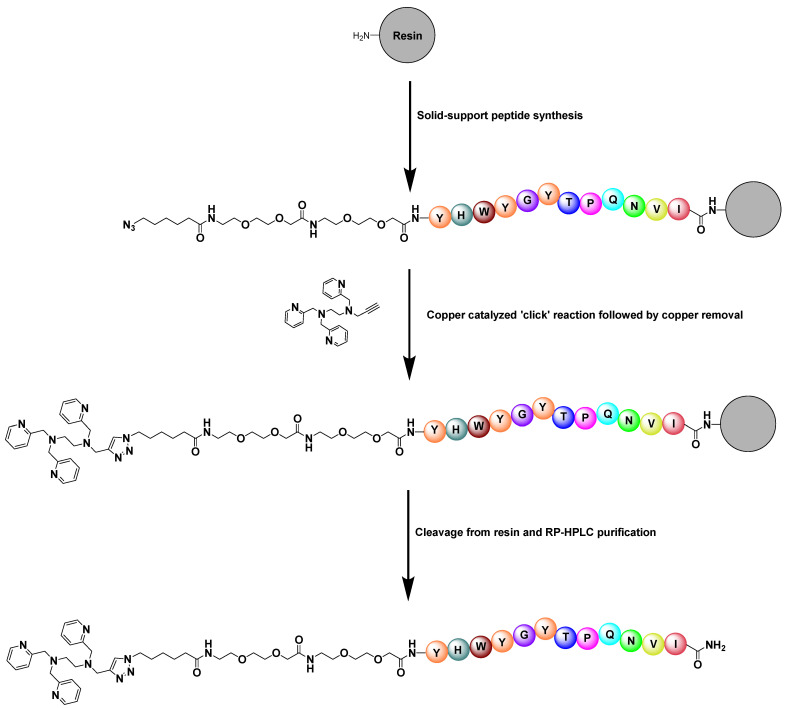
Outline of the synthesis of TZTPEN-GE11.

**Figure 2:**
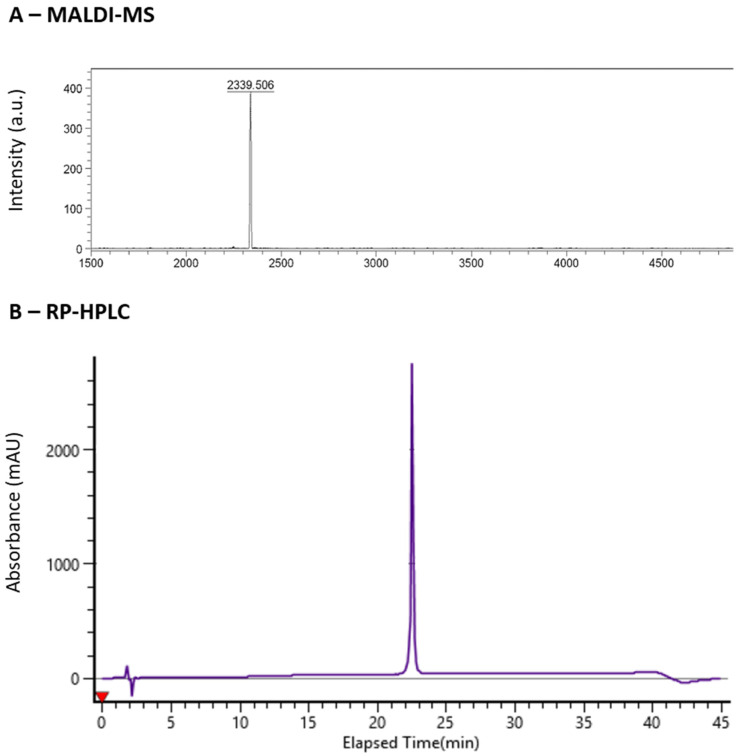
(**A**) Maldi-MS spectra of TZTPEN-GE11 (calc. mass: 2339.16; found mass: 2339.50) and (**B**) final RP-HPLC analysis of TZTPEN-GE11. Detection wavelength 214 nm. The red triangle (left corner) indicates starting point of UV/Vis-spectra.

An azido-modified GE11 peptide was synthesized using standard Fmoc-protected amino acid (AA) building blocks, except for the azido-modified linker situated in the N-terminal of the peptide (Scheme 1). Initially, Fmoc-8-amino-3,6-dioxaoctanoic acid (Fmoc-O2Oc-OH) was incorporated twice consecutively, from the C- to the N-termini, with the standard microwave-assisted method provided by the CEM Liberty Blue machine. These steps were immediately followed by the incorporation of the 6-azido-caproic acid (N3-Hx-OH) building block. The dioxaoctanoic acid moiety enabled precise control over the linker length, with PEG2 selected for its ease of synthesis and maintained hydrophilicity [45,46]. Previous studies have shown that PEG linker length does not compromise peptidase stability [34]. The GE11 peptide was modified at the C-terminus with an amide group to enhance stability and potentially improve cellular uptake [47,48]. While this modification might reduce solubility due to the absence of a carboxylate group, the PEG linker effectively mitigated this limitation. The synthesis of the azido-modified GE11 peptide was carried out on a Rink amide resin, which facilitates efficient cleavage and yields a peptide with an amide-modified C-terminus. Click chemistry was employed to conjugate the peptide to the amino-pyridyl chelator due to its robustness and suitability for forming stable triazole linkages, which is widely utilized in drug discovery [49]. The CuAAC click reaction between the resin-bound azide-conjugated peptide and the alkylated chelate precursor, N1-(prop-2-yn-1-yl)-N1,N2,N2-tris(pyridin-2-ylmethyl)ethane-1,2-diamine (TPENYNE), was performed using a modified version of a previously described protocol [50]. Under an inert atmosphere, a syringe containing the reaction mixture fitted with a filter and a stopper was rotated at room temperature for 6 h, allowing the resin to be thoroughly mixed. Upon completion, the resin was washed thoroughly with H_2_O, dimethyl sulfoxide (DMSO), dichloromethane (DCM), and a mixture of TPENYNE and EDTA in H_2_O:DMSO; 1:1; V;V in order to remove any copper residue from the CuAAC reaction. Complete removal of copper was confirmed by the disappearance of the green coloration from the resin. Cleavage of the peptide from the resin was achieved with trifluoroacetic acid (TFA), using H_2_O and triisopropylsilane (TIPS) as scavengers [51]. The crude peptide-chelator conjugate was purified twice using reversed-phase high-performance liquid chromatography (RP-HPLC) (Figure 2A). TZTPEN-GE11 was ultimately isolated in a 60% yield as a white solid, and the MALDI-MS spectra (Figure 2B) confirm high purity.

### 2.2. Radiolabeling and Quality Control

#### 2.2.1. [^57^Co]Co-TZTPEN-GE11

**Figure 3:**
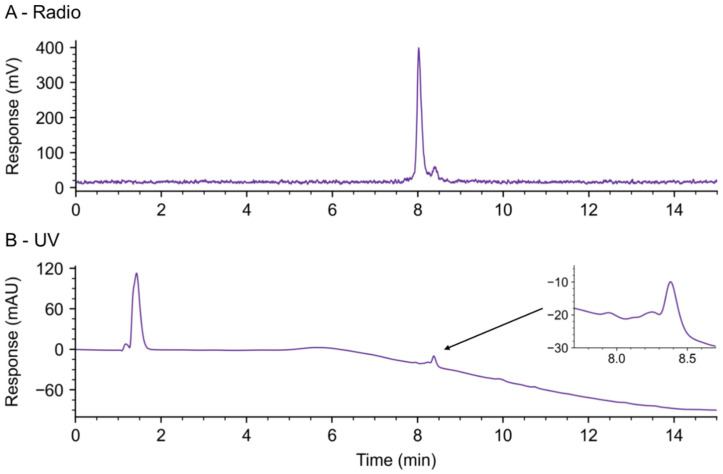
Radio chromatogram (**A**) and UV chromatogram (**B**) following analytical RP-HPLC of TZTPEN-GE11 radiolabeled with ^57^Co (0.29 MBq/nmol). Detection wavelength 220 nm.

TZTPEN-C6-Peg2-GE11 was readily radiolabeled with ^57^Co, using [^57^Co]CoCl_2_ at pH 5.5 to form [^57^Co]Co-TZTPEN-GE11, with a radiochemical yield of 99%, as confirmed using instant thin layer chromatography (ITLC) and RP-HPLC analysis (Figure 3). The radio-chromatogram displayed a major peak at approximately 8.02 min and a minor peak at 8.40 min, accounting for 88% and 12% of the radioactivity, respectively. Variability in the ratio between the percentages of these two peaks was observed across samples, averaging 83 ± 11%:17 ± 11% over eight radiolabeling experiments performed under comparable conditions. Four peaks were observed in the UV chromatogram, eluting at 7.96, 8.13, 8.25, and 8.38 min, and corresponding to the same timeframe as the radio peaks. The UV chromatogram of unlabeled TZTPEN-GE11 (Figure S1) also showed several small shoulders and peaks at the same times, although less distinguishable, likely due to saturation by the taller and broader main peak. These align with the peaks in Figure 3. The occurrence of multiple peaks might result from TZTPEN-GE11 folding into various conformations and/or contamination with cold metal isotopes.

The 1,2,3-triazole functional group of TZTPEN-GE11 can be expected to act as a metal donor since it is part of a multidentate chelating ligand [52,53]. Co^2+^ and Co^3+^ will favor octahedral six-coordination for this type of ligand, and this is observed for the Co^2+^ and Co^3+^ complexes of the very closely related hexadentate aminopyridyl ligand TPEN [54] that was mentioned in the introduction and where the only difference is a fourth pyridyl donor in place of the triazole group. A reasonable rationalization for the two radiolabeled peaks is the presence of the Co complex in both the +2 and +3 oxidation states. The different overall charges and possible effects on conformation give rise to two peaks. The redox potential of the Co complex of TZTPEN can be expected to be similar to the redox potential of [Co(TPEN)]^3+/2+^, which is 0.497 V vs. NHE at pH 7 [55]. On this basis, we assume that the major peak at 8.02 min is due to Co-TZTPEN-GE11 with the Co in the +2 state. This is probably not ideal, given the expected lability of the Co^2+^ vs. Co^3+^ state. The ratio between the two peak areas remained similar for up to 72 h when performing stability studies in PBS (pH 5), with the first peak maintaining its prominence. No release of ^57^Co was observed (Figure S2), suggesting high stability with radiochemical purity of >99% under these conditions. For subsequent in vitro and in vivo studies, both peaks were considered to constitute the radiochemically pure [^57^Co]Co-TZTPEN-GE11.

#### 2.2.2. Cellular Uptake

**Figure 4:**
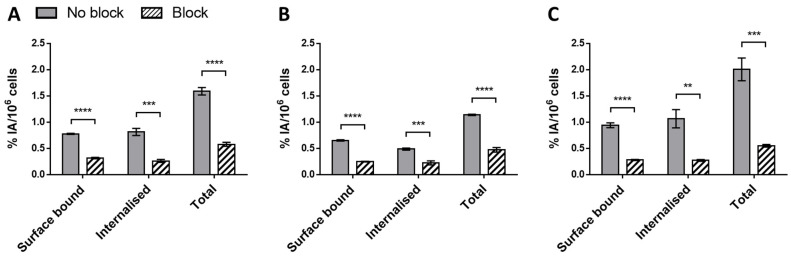
Cellular uptake of [^57^Co]Co-TZTPEN-GE11 (2.5 pmol/well) in (**A**) PC3, (**B**) U87, and (**C**) A431 cells without block (grey bars) or with 1000 × unlabeled TZTPEN-GE11 as block (striped bars) 1 h after addition of [^57^Co]Co-TZTPEN-GE11. Data are expressed as percentage injected activity (IA) per 10^6^ cells and are presented as mean ± SD (*n* = 3). Unpaired Student’s t test was performed on the data to determine significant differences between when the block was present and when it was not. ** *p* ≤ 0.01, *** *p* ≤ 0.001, **** *p* ≤ 0.0001.

Uptake studies of [^57^Co]Co-TZTPEN-GE11, in the presence or absence of a 1000-fold excess of unlabeled TZTPEN-GE11 (as an EGFR blocking agent), were performed in the human prostate adenocarcinoma cell line PC3, the human glioblastoma cell line U87, and in the human carcinoma cell line A431. The A431 cells were chosen as a positive control due to their high EGFR expression [56]. Cellular uptake could be significantly blocked in all three cell lines, indicating that [^57^Co]Co-TZTPEN-GE11 shows EGFR-specific uptake. The highest specific uptake (cellular uptake minus cellular uptake in the presence of block) was observed in A431 cells (1.45 ± 0.22%), followed by PC3 cells (1.01 ± 0.08%) and U87 cells (0.66 ± 0.05%) (Figure 4), confirming that cellular uptake was mediated by the GE11 targeting moiety. We note that the specific cellular uptake was higher in both PC3 and U87 cells compared with the values reported for the uptake of GE11 combined with an amide-coupled [^18^F]fluoropropionate ([^18^F]FP-lys-GE11), where values of 0.33% and 0.32%, respectively, were measured after the same incubation time (1 h) [30]. No specific binding in A431 cells for a ^68^Ga labeled GE11-conjugate of 1,4,7-triazacyclononane,1-glutaric acid-4,7-acetic acid (NODAGA) was observed [34].

#### 2.2.3. Ex Vivo Biodistribution and SPECT/CT Imaging

**Figure 5:**
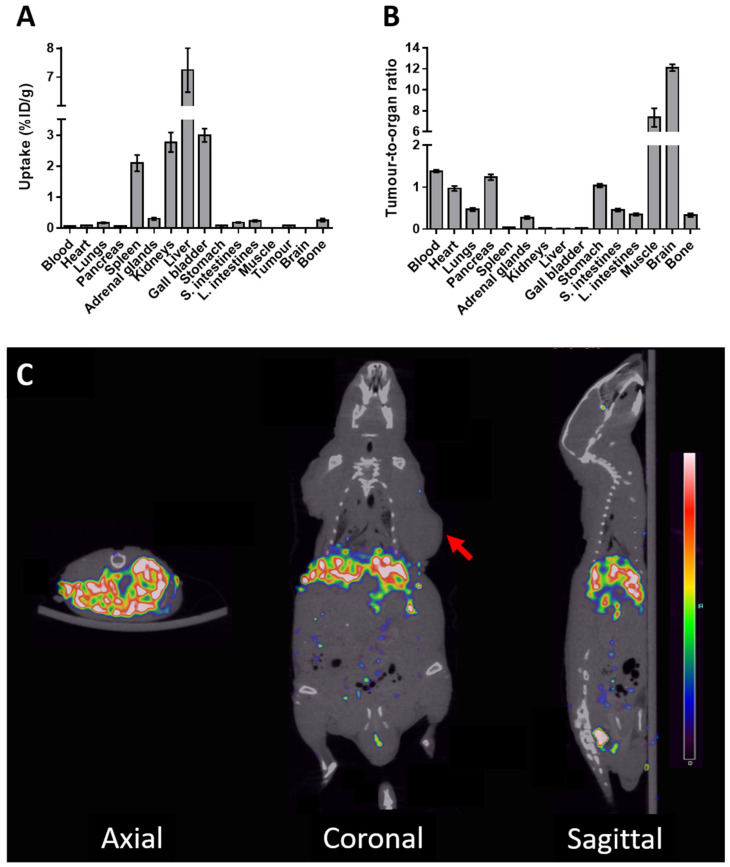
(**A**) Ex vivo biodistribution expressed as percent injected dose/g tissue (%ID/g) and (**B**) tumor-to-organ ratios of [^57^Co]Co-TZTPEN-GE11 (41 ± 5 kBq, 0.34 MBq/nmol) in PC3 xenograft mice 2 h post-injection (*n* = 3). Data are presented as mean ± SEM. (**C**) SPECT/CT of a PC3 xenograft mouse injected with [^57^Co]Co-TZTPEN-GE11 (0.34 MBq, 0.34 MBq/nmol), sacrificed, and scanned 2 h post-injection. The arrow indicates the tumor.

The EGFR-specific uptake in vitro prompted our investigation of the tumor uptake and biodistribution of [^57^Co]Co-TZTPEN-GE11 in PC3 tumor-bearing mice. Ex vivo biodistribution and SPECT/CT imaging were performed 2 h post-injection (PI) of [^57^Co]Co-TZTPEN-GE11. The highest uptake per gram tissue was observed in the liver (7.3 ± 0.8%ID/g), followed by the gall bladder (3.0 ± 0.2%ID/g), the kidneys (2.8 ± 0.3%ID/g), and the spleen (2.1 ± 0.3%ID/g) (Figure 5A). Similarly, the SPECT/CT scans (Figure 5C) also showed the highest uptake in the kidneys and liver. The total harvested activity in the ex vivo biodistribution studies was measured to be 13.6 ± 1.7% of the administered activity. Thus, most of the administered activity remained unaccounted for. [^57^Co]Co-TZTPEN-GE11 was thus presumed to have been excreted through the kidneys with urine and partly by hepatobiliary excretion, as indicated by the high uptake in the gall bladder. Although displaying tumor-to-blood and tumor-to-muscle ratios of 1.38 ± 0.03% and 7.34 ± 0.88% (Figure 5B), the biodistribution of [^57^Co]Co-TZTPEN-GE11 showed poor tumor uptake (0.08 ± 0.01% ID/g), and no tumor uptake could be observed in the SPECT/CT scan. For comparison, Li et al. [30] reported the uptake of ^18^F-labeled GE11-lysine compound to be 5.85% ID/g 30 min PI in SMMC-7721 tumor-bearing mice, Chen et al. [28] observed a 2.03% ID/g uptake of ^68^Ga-labeled NOTA-GE11 complex 2 h PI in NCI-H292 tumor-bearing mice, and Rahmanian et al. [31] observed uptake of 2.33% ID/g of ^99m^Tc-labeled tricine-HYNIC-SSS-GE11 1 h PI in SKOV-3 bearing mice. Although different cell lines were used for inoculation in these studies compared with our work, the lack of tumor uptake of [^57^Co]Co-TZTPEN-GE11 in vivo stands in contrast to the in vitro results we obtained. This could be due to several factors: (i) release of cobalt, (ii) low radioligand stability in vivo, or (iii) a low affinity of GE11 toward EGFR. The uptake of activity in the bone could indicate the release of cobalt from the [^57^Co]Co-TZTPEN-GE11 since the biodistribution of [^55^Co]CoCl_2_ reported in mice, demonstrated high uptake in the liver, kidneys, large intestines, and heart, as well as bone [8,57], although a low uptake in the heart was observed in the current study. This is consistent with our assumption mentioned above that most of the [^57^Co]Co-TZTPEN-GE11 is in the Co^2+^ state. The consequence will be a greater degree of metal decoordination compared with a more robust Co^3+^ system, for example, a [Co(NOTA)]^−^, which shows a redox potential of −0.07 V vs. NHE [58] over half a volt lower. However, low in vivo stability and low affinity toward EGFR, as observed by Striese et al. [36], who investigated two ^64^Cu-labeled GE11 analogs, are presumably contributing factors to the low tumor uptake.

## 3. Materials and Methods

### 3.1. Synthesis and Characterisation

*N*,*N*,*N*-tris(pyridin-2-ylmethyl)ethane-1,2-diamine was synthesized according to a literature procedure [58]. ESI-MS spectra were recorded on a nano spray Bruker micro-OTOF-Q II in positive ionization mode, and mMass version 5.5.0 was used for the visualization of the obtained data. IR spectra were recorded on an Agilent Cary 603 FTIR. Peptide synthesis was carried out on a CEM Liberty BlueTM peptide synthesizer. RP-HPLC purification and analysis of the TZTPEN-GE11 peptide was performed on a Waters AutoPurification™ System. MS-MALDI of the final TZTPEN-GE11 peptide was recorded on a Bruker Daltonics Microflex LT MAIDI-TOF MS instrument (Burker, Billerica, MA, USA).

*N*-(prop-2-yn-1-yl)-*N*,*N*,*N*-tris(pyridin-2-ylmethyl)ethane-1,2-diamine (TPENYNE) was synthesized using a modified literature procedure [59]. Briefly, *N*,*N*,*N*-tris(pyridin-2-ylme-thyl)ethane-1,2-diamine (0.505 g, 1.51 mmol) and K_2_CO_3_ (0.874 g, 6.46 mmol) were dissolved in anhydrous THF (50 mL) and cooled to 0 °C. Propargyl bromide (80% in toluene) (0.232 g, 1.56 mmol, 0.12 mL) diluted in anhydrous THF (10 mL) was then added dropwise (30 min). The reaction mixture was slowly heated to ambient temperature and stirred for 16 h. After this time, it was filtered through a K_2_CO_3_ pad before the solvent was removed in vacuo. The resulting oil was stored under reduced pressure for 48 h to afford TPENYNE as a brown oil (yield 82%, 0.465 g, 0.125 mmol). ^1^H-NMR (400 MHz, DMSO-d6), δ (ppm): 2.63 (m, 4H, NC*H*_2_), 3.13 (t, 1H, J = 2.4 Hz, C≡C*H*), 3.34 (d, 2H, J = 2.4 Hz, C*H*_2_C≡CH), 3.67 (s, 2H, NC*H*_2_), 3.73 (s, 4H, NC*H*_2_), 7.22 (m, 3H, 3 × py*H*), 7.36 (d, 1H, J = 7.6 Hz, py*H*), 7.49 (td, 2H, J = 7.6, 1.8 Hz, py*H*), 7.68 (td, 1H, J = 7.6, 1.8 Hz, py*H*), 7.73 (m, 2H, 2 × py*H*), 8.52 (m, 3H, 3 × py*H*). ^13^C-NMR (100 MHz, DMSO-d6), δ (ppm): 159.3, 158.9, 158.3, 149.67, 149.65, 136.3, 122.5, 122.4, 122.04, 121.9, 78.9, 75.6, 66.9, 59.9, 59.4, 51.4, 50.6, 25.1. ESI-MS (pos. mode, MeCN): *m*/*z* 372.2182 (100%, [C_23_H_26_N_5_]^+^ calcd. 372.2183), 226.1329 (55%, [C_14_H_16_N_3_]^+^ calcd. 817.0748).

### 3.2. GE11-TZTPEN

The GE11 peptide was assembled using a fully automated and microwave-assisted Fmoc-solid phase peptide synthesis (SPPS) procedure in a CEM Liberty BlueTM peptide synthesizer. The synthesis was carried out on a TentaGel^®^ XV—RAM (Rapp Polymere) Resin (100–200 µm) 0.23 mmol/g resin (Iris Biotech) using a 0.1 mmol scale. The RAM-solid support allowed for the C-terminal to be amide-protected after cleavage. Amino acid building blocks were incorporated through alternating cycles of amino acid (AA) coupling and Fmoc deprotection. *N*,*N′*-diisopropylcarbodiimide (DIC, 1 M in DMF) and ethyl (2E)-2-cyano-2-hydroxyiminoacetate (Oxyma, 1 M in DMF) were used as activator and activator base solutions for AA couplings, while 20% piperidine in DMF was used in the fmoc deprotection steps. The PEG linker between the GE11 peptide and the metal binding chelator was constructed by incorporating Fmoc-O2Oc-OH (Iris Biotech) in succession, followed by the incorporation of N3-Hx-OH (Iris Biotech) through the above-mentioned protocol. After synthesis, the solid support containing the peptide was washed with DMF(×4) and DCM(×5) and used without any further purification for the ‘click’ reaction step.

The loading of the resin and the quantity of the peptide were assumed to be equivalent; thus, 0.028 mmol of resin was transferred to a syringe fitted with a filter and a stopper before it was soaked in ^t^BuOH:H_2_O (2:3; V:V) for 1 h. The stopper was removed to drain any excess solution and refitted before the TPENYNE (0.4 M: 70 µL) dissolved in ^t^BuOH was added to the resin, followed by 1 mL of ^t^BuOH:H_2_O (2:3; V:V) solution (purged with argon). The coupling solution was simultaneously prepared separately. First, CuSO_4_ (1 M; 58 µL) dissolved in H_2_O was added to a vial before tris(3-hydroxypropyltriazolylmethyl)amine (THPTA) (0.16 M, 875 µL) dissolved in ^t^BuOH was added, followed by sodium ascorbate dissolved in H_2_O (1 M; 140 µL) and then 550 µL of H_2_O. The coupling solution was purged with argon before it was transferred to the resin-alkyne solution. *N*,*N-*diisopropylethylamine (DIPEA) (10 µL) was added, and the syringe was flushed with argon, sealed and gently rotated for 6 h. The syringe was placed above a beaker, and the stopper was removed to drain the excess solution from the syringe. The resin remained blue/green after washing through with 5 × DMSO, 5 × H_2_O and 5 × DCM and 3 × EDTA (5 M) aqueous solution. Finally, a mixture of EDTA and TPENYNE in a 1:1 ratio (0.1 M; H_2_O:DMSO; 1:1; V:V) allowed for the complete removal of copper contamination derived from the CuAAC reaction. This was evidenced by the liquid coming out of the syringe being blue/green and the resin becoming yellow. This washing step was repeated five times before the resin was washed with 3 × H_2_O, 3 × DMF, and 3 × DCM. TZTPEN-GE11 was cleaved from the resin using 95% trifluoroacetic acid (TFA)/2.5% H_2_O/2.5% triisopropylsilane (TIPS) with a 1.5 h reaction time. The TFA was reduced under nitrogen flow before the peptide was precipitated in cold diethyl ether.

RP-HPLC analysis (Figure 2B) was carried out on a Waters AutoPurification™ System (Waters corporation, Milford, MA, USA) connected through a Waters 2545 Quaternary Gradient Module, Waters 2489 UV/Visible detector, and a Waters 2707 Autosampler that had been equipped with a Zorbax Eclipse XDB-C18 Column (4.6 × 150 mm 5-Micron, Agilent Technologies, Santa Clara, CA USA). Elution was performed starting with an isocratic hold of A-/B-buffer (95/5, *v*/*v*) for 3 min, followed by a linear gradient to 80% B-buffer over 32 min at a flow rate of 1.0 mL/min [A: 99.9% Milli Q water and 0.1% TFA; B: 89.95% acetonitrile, 9.95% Milli Q water and 0.1% TFA].

RP-HPLC purification was carried out on the same Waters system (Waters corporation, Milford, MA, USA) fitted with a Zorbax Eclipse Plus C18 (21.2 × 150 mm 5-Micron, Agilent Technologies, Santa Clara, CA USA) semi-preparative column. TZTPEN-GE11 was purified twice on a semi-preparative column using two different methods. The first purification was performed by eluting from an isocratic hold of A-/B-buffer (95/5, *v*/*v*) for 3 min, followed by a linear gradient to 80% B-buffer over 32 min at a flow rate of 5.0 mL/min [A: 99.9% Milli Q water and 0.1% TFA; B: 89.95% acetonitrile, 9.95% Milli Q water and 0.1% TFA]. The second purification was performed using an elution, starting with an isocratic hold of A-/B-buffer (95/5, *v*/*v*) for 3 min, followed by a linear gradient to 80% B-buffer over 42 min at a flow rate of 5.0 mL/min [A: 99.9% Milli Q water and 0.1% TFA; B: 89.95% acetonitrile, 9.95% Milli Q water and 0.1% TFA]. The mass of the peptide was found using a Bruker Daltonics Microflex LT MALDI-TOF MS instrument (Burker, Billerica, MA, USA) (Figure 2A). The final yield of the purified TZTPEN-GE11 peptide was 40 mg (60%: 0.017 mmol) based on the loading of the resin.

### 3.3. Radiolabeling and Quality Control

Radiolabeling of TZTPEN-GE11 was performed by adding 5–20 µL [^57^Co]CoCl_2_ (1.34–2.75 MBq, 0.1 M HCl) from a stock solution (99.906%, JSC Isotope, Moscow, Russia), 5–20 µL 0.2 M sodium acetate buffer pH 5.55 (NaOAc), 89–97 µL MQ water, and 3–4 µL 1 nmol/µL TZTPEN-GE11 dissolved in DMSO:MQ (1:19) to a HPLC vial. The vial was sealed and heated for 10 min at 60 °C by microwave irradiation. The radiochemical yield was determined using ITLC, which was performed on Biodex strips (150–771, Biodex Medical Systems, Shirley, NY, USA) in 0.2 M citric acid (pH 2.0). After elution, the TLC strips were cut at the line, and each part was counted on a 2470 Wizard Automatic Gamma Counter (PerkinElmer). Radiochemical purity (RCP) of the radiolabeled complex was determined using analytical RP-HPLC performed on a Hitachi LaChrom Elite system with diode array detection (220 nm) in series with radio-detection and a Phenomenex LUNA C18 column (125 × 4 mm, 5 µm) at 1 mL/min. HPLC-analysis was performed with a gradient program (A:B, MQ water with 0.1% trifluoroacetic acid (TFA)–acetonitrile): 0–2 min 90% A, followed by 90–20% A from 2 to 12 min, then 20% A from 12–14 min, and lastly, 20–90% A from 14–15 min.

### 3.4. Stability Studies

[^57^Co]Co-TZTPEN-GE11 was prepared as described above (2.4 ± 0.3 MBq, 1.22 ± 0.14 MBq/nmol, *n* = 3) and added to 600 µL phosphate-buffered saline (PBS), yielding a solution with a pH of 5. The mixture was incubated at 37 °C for 72 h. Aliquots of 40 µL were collected after 0, 24, 48, and 72 h. Collected samples were analyzed using RP-HPLC to determine the radiochemical purity, as described above. The radiochemical purity of [^57^Co]Co-TZTPEN-GE11 prior to dilution in PBS was also analyzed using RP-HPLC.

### 3.5. Octanol/PBS Partition Coefficient (LogP)

LogP determination was performed by diluting 3 µL of [^57^Co]Co-TZTPEN-GE11 (15–41 kBq, 1.13–1.35 MBq/nmol, *n* = 3) in 503 µL PBS (pH 7.4). A total of 500 µL of the solution was added to 500 µL 1-octanol, preconditioned with PBS (*n* = 3). The mixture was vortexed for 10 min and then centrifuged for 5 min (6000 rpm) at room temperature, after which the two phases were isolated. Aliquots of 100 µL were collected from each phase, and the activity was measured on a 2470 Wizard Automatic Gamma Counter (PerkinElmer, Waltham, MA, USA), from which the logP values were calculated.

### 3.6. In Vitro Studies

#### 3.6.1. Cell Lines

PC3 (human prostate adenocarcinoma) was obtained from the American Type Culture Collection (CRL-1435, ATCC, Manassas, VA, USA). The cells were grown in Roswell Park Memorial Institute 1640 supplemented with 10% fetal bovine serum (FBS) and 1% penicillin/streptomycin (P/S) (all from ThermoFisher, Roskilde, Denmark). U87 (human glioblastoma) was obtained from the European Collection of Authenticated Cell Cultures. The cells were grown in Dulbecco’s Modified Eagle Medium (DMEM) (Merck, Darmstadt, Germany) supplemented with 10% FBS, 1% P/S, 2 mM GlutaMAX (ThermoFischer, Roskilde, Denmark), and 1 mM sodium pyruvate (ThermoFischer, Roskilde, Denmark). A431 (human epidermoid carcinoma cells) were obtained from Cell Line Services (Eppelheim, Germany). The cells were grown in DMEM supplemented with 10% FBS and 1% P/S. All cells were maintained at 37 °C in a humidified atmosphere with 5% CO_2_. All in vitro studies were performed in technical triplicates.

#### 3.6.2. Subcellular Distribution 

For all cell lines, 4 × 10^5^ cells were seeded per well in six-well plates and incubated at 37 °C in a humidified atmosphere with 5% CO_2_. The following day, cells were washed once with PBS and incubated with 0.9 kBq [^57^Co]Co-TZTPEN-GE11 (2.5 pmol) per well in DMEM supplemented with 1% bovine serum albumin (BSA, Sigma) at 37 °C in a humidified atmosphere with 5% CO_2_. After 1 h, the incubation medium was removed, and the cells were washed three times with cold PBS. Surface-bound activity was collected after 5 min of incubation with cold acid wash (0.2 M NaOAc, 0.5 M NaCl pH 2.5, 4 °C). Cells were solubilized using 1 M NaOH. Collected fractions (surface-bound and internalized) were measured on a gamma counter. Non-specific binding was assessed through co-incubation with 2.5 nmol TZTPEN-GE11. At the same time, six-well plates with 4 × 10^5^ cells/well were seeded on the first day and counted on the experiment day to normalize the percentage of fractions per 1 × 10^6^ cells at incubation time (counting controls). Uptake was normalized to 10^6^ cells and presented as a percentage of total added activity per 10^6^ cells.

#### 3.6.3. Ex Vivo Biodistribution and SPECT/CT Imaging of Tumor-Bearing Mice

Four 8-week-old male BALB/c nude mice (Janvier) were subcutaneously inoculated with 10 × 10^6^ PC3 cells dissolved in an extracellular matrix (Merck, Darmstadt, Germany) in the shoulder. Twenty-three days later, when all mice had a visible tumor that reached between 5–8 mm in length, the animals were anesthetized with a mixture of 1.5–2% isoflurane and 100% oxygen and placed on a heating pad (37 °C), and [^57^Co]Co-TZTPEN-GE11 was injected in the tail vein. The animal used for SPECT/CT imaging was injected with 200 µL [^57^Co]Co-TZTPEN-GE11 diluted in PBS containing 0.1% BSA (0.34 MBq, 0.34 MBq/nmol). The animals used for ex vivo biodistribution were injected with 150 µL [^57^Co]Co-TZTPEN-GE11 in PBS (41 ± 5 kBq, 0.34 MBq/nmol). After two hours, the animals were euthanized by cervical dislocation.

For the ex vivo biodistribution, organs were collected and weighed, and the amount of activity in each organ was measured on a 2470 Wizard Automatic Gamma Counter (PerkinElmer). The uptake in the organs was then calculated as the percentage of injected activity per gram of tissue (%ID/g). Small-animal SPECT/CT scanning was performed on a Siemens INVEON multimodality pre-clinical scanner (Siemens Pre-clinical Solutions, Knoxville, TN, USA). The mouse was placed feet first in a prone position on a dedicated SPECT/CT mouse bed (38 mm). First, a two-bed whole-body CT scan was performed to determine anatomic orientation and attenuation correction. The CT was performed with the following settings: half rotation, 180 projections, 4 × 4 bin, and low magnification covering a scan length of 95 mm. The exposure settings were 80 kV and 500 µA at 350 ms. The CT scan was reconstructed using the Feldkamp algorithm, with a Shepp-Logan filter, slight noise reduction, and Hounsfield calibration. The CT scan was followed by a SPECT scan obtained, with a factory standard energy window set for ^57^Co (photopeak 110–134 keV), using two synchronized whole-body mouse collimators (MWB-1.0). The acquisition parameters included a 30 mm radius of rotation, 60 projections/detector, 1.0 revolution, 6° angle, and a 44 mm bed travel, providing an axial scan length of 110 mm. The acquisition was performed with 900 sec/step, resulting in a final SPECT acquisition time of 15 h. CT and SPECT images were co-registered using a transformation matrix, and the SPECT data were reconstructed using a MAP3D algorithm (matrix 128 × 128, 16 iterations, and six subsets) with a maximum resolution of 1.3 mm. SPECT/CT data were analyzed using the dedicated imaging analysis software Inveon Research Workplace (https://www.inyeon.ai/community, accessed on 7 December 2024, Siemens Medical Solutions Malvern, PA, USA).

### 3.7. Statistics

All data are presented as mean ± SD or SEM, as stated in the figure legend. Statistical analysis included an unpaired *t* test for comparison of cellular uptake with or without excess unlabeled TZTPEN-GE11 unlabeled TZTPEN-GE11 as a blocking agent. All statistical analyses were performed using Graphpad Prism 6. Statistically significant values with increasing significance were labeled as follows: * *p* ≤ 0.05, ** *p* ≤ 0.01, *** *p* ≤ 0.001, **** *p* ≤ 0.0001.

## 4. Conclusions

Although multidentate chelating amino-polypyridyl ligands are commonly used in coordination chemistry and well known to form robust cobalt complexes, this class of chelator had not previously been applied to nuclear medicine nor conjugated to a peptide. We designed a synthetic protocol to make this possible by clicking an alkylated TPEN-type chelator to an azido-modified linker, which was, in turn, attached to the EGFR-targeting peptide GE11. TZTPEN-GE11 is readily labeled with ^57^Co in 99% radiochemical yield, and although specific uptake was measurable in cancer cells, a high off-target accumulation in the liver, gall bladder, spleen, kidneys, and bone, along with the lack of tumor uptake in PC3 xenograft mice, leads us to conclude that this construct is not suitable for delivery of cobalt radiopharmaceuticals.

## Data Availability

The raw data supporting the conclusions of this article will be made available by the authors on request.

## Supplementary Materials

The following supporting information can be downloaded at https://www.mdpi.com/article/10.3390/molecules30020212/s1. Figure S1: UV chromatogram following analytical RP-HPLC of TZTPEN-GE11 dissolved in DMSO and diluted in MQ water (1:19). Two peaks are observed: one at 1.48 min and one at 8.22 min with a small shoulder present at approximately 0.15 min before the main peak; Figure S2: Stability of [^57^Co]Co-TPEN-GE11 (1.13-1.38 MBq/nmol, RCY > 98.92%) in PBS for up to 72 h, at pH 5. The radiochemical purity was determined by RP-HPLC analysis. Data is presented as Mean ± SD, n = 3.

## References

[1] Andersen T.L., Baun C., Olsen B.B., Dam J.H., Thisgaard H. (2020). Improving Contrast and Detectability: Imaging with [^55^Co]Co-DOTATATE in Comparison with [^64^Cu]Cu-DOTATATE and [^68^Ga]Ga-DOTATATE. J. Nucl. Med..

[2] Baun C., Dam J.H., Hildebrandt M.G., Ewald J.D., Kristensen B.W., Gammelsrød V.S., Olsen B.B., Thisgaard H. (2023). Preclinical evaluation of [^58m^Co]Co-DOTA-PSMA-617 for Auger electron therapy of prostate cancer. Sci. Rep..

[3] Baun C., Mitran B., Rinne S.S., Dam J.H., Olsen B.B., Tolmachev V., Orlova A., Thisgaard H. (2020). Preclinical Evaluation of the Copper-64 Labeled GRPR-Antagonist RM26 in Comparison with the Cobalt-55 Labeled Counterpart for PET-Imaging of Prostate Cancer. Molecules.

[4] Dam J.H., Olsen B.B., Baun C., Høilund-Carlsen P.F., Thisgaard H. (2016). In Vivo Evaluation of a Bombesin Analogue Labeled with Ga-68 and Co-55/57. Mol. Imaging Biol..

[5] Dam J.H., Olsen B.B., Baun C., Høilund-Carlsen P.F., Thisgaard H. (2017). A PSMA Ligand Labeled with Cobalt-55 for PET Imaging of Prostate Cancer. Mol. Imaging Biol..

[6] Goethals P., Volkaert A., Vandewielle C., Dierckx R., Lameire N. (2000). 55Co-EDTA for renal imaging using positron emission tomography (PET): A feasibility study. Nucl. Med. Biol..

[7] Houson H.A., Tekin V., Lin W., Aluicio-Sarduy E., Engle J.W., Lapi S.E. (2022). PET Imaging of the Neurotensin Targeting Peptide NOTA-NT-20.3 Using Cobalt-55, Copper-64 and Gallium-68. Pharmaceutics.

[8] Mastren T., Marquez B.V., Sultan D.E., Bollinger E., Eisenbeis P., Voller T., Lapi S.E. (2015). Cyclotron Production of High-Specific Activity 55Co and In Vivo Evaluation of the Stability of 55Co Metal-Chelate-Peptide Complexes. Mol. Imaging.

[9] Radford L.L., Fernandez S., Beacham R., El Sayed R., Farkas R., Benešová M., Müller C., Lapi S.E. (2019). New ^55^Co-labeled Albumin-Binding Folate Derivatives as Potential PET Agents for Folate Receptor Imaging. Pharmaceuticals.

[10] Thisgaard H., Olesen M.L., Dam J.H. (2011). Radiosynthesis of ^55^Co- and ^58m^Co-labelled DOTATOC for positron emission tomography imaging and targeted radionuclide therapy. J. Label. Compd. Radiopharm..

[11] Thisgaard H., Olsen B.B., Dam J.H., Bollen P., Mollenhauer J., Høilund-Carlsen P.F. (2014). Evaluation of cobalt-labeled octreotide analogs for molecular imaging and auger electron-based radionuclide therapy. J. Nucl. Med..

[12] Uusijärvi H., Bernhardt P., Ericsson T., Forssell-Aronsson E. (2006). Dosimetric characterization of radionuclides for systemic tumor therapy: Influence of particle range, photon emission, and subcellular distribution. Med. Phys..

[13] Anderegg G., Hubmann E., Podder N.G., Wenk F. (1977). Pyridinderivate als Komplexbildner. XI. Die Thermodynamik der Metallkomplexbildung mit Bis-, Tris- und Tetrakis[(2-pyridyl)methyl]-aminen. Helv. Chim. Acta.

[14] Schwarzenbach G., Freitag E. (1951). Komplexone XX. Stabilitätskonstanten von Schwermetallkomplexen der Äthylendiamin-tetraessigsäure. Helv. Chim. Acta.

[15] Brennan C.W., Verhaak R.G., McKenna A., Campos B., Noushmehr H., Salama S.R., Zheng S., Chakravarty D., Sanborn J.Z., Berman S.H. (2013). The somatic genomic landscape of glioblastoma. Cell.

[16] Day K.C., Hiles G.L., Kozminsky M., Dawsey S.J., Paul A., Broses L.J., Shah R., Kunja L.P., Hall C., Palanisamy N. (2017). HER2 and EGFR Overexpression Support Metastatic Progression of Prostate Cancer to Bone. Cancer Res..

[17] Di Lorenzo G., Tortora G., D’Armiento F.P., De Rosa G., Staibano S., Autorino R., D’Armiento M., De Laurentiis M., De Placido S., Catalano G. (2002). Expression of Epidermal Growth Factor Receptor Correlates with Disease Relapse and Progression to Androgen-independence in Human Prostate Cancer1. Clin. Cancer Res..

[18] Libermann T.A., Nusbaum H.R., Razon N., Kris R., Lax I., Soreq H., Whittle N., Waterfield M.D., Ullrich A., Schlessinger J. (1985). Amplification, enhanced expression and possible rearrangement of EGF receptor gene in primary human brain tumours of glial origin. Nature.

[19] Mitsudomi T., Yatabe Y. (2010). Epidermal growth factor receptor in relation to tumor development: EGFR gene and cancer. FEBS J..

[20] Nastały P., Stoupiec S., Popęda M., Smentoch J., Schlomm T., Morrissey C., Żaczek A.J., Beyer B., Tennstedt P., Graefen M. (2020). EGFR as a stable marker of prostate cancer dissemination to bones. Br. J. Cancer.

[21] Schlegel J., Merdes A., Stumm G., Albert F.K., Forsting M., Hynes N., Kiessling M. (1994). Amplification of the epidermal-growth-factor-receptor gene correlates with different growth behaviour in human glioblastoma. Int. J. Cancer.

[22] Schlomm T., Kirstein P., Iwers L., Daniel B., Steuber T., Walz J., Chun F.H.K., Haese A., Kollermann J., Graefen M. (2007). Clinical Significance of Epidermal Growth Factor Receptor Protein Overexpression and Gene Copy Number Gains in Prostate Cancer. Clin. Cancer Res..

[23] Li Z., Zhao R., Wu X., Sun Y., Yao M., Li J., Xu Y., Gu J. (2005). Identification and characterization of a novel peptide ligand of epidermal growth factor receptor for targeted delivery of therapeutics. FASEB J..

[24] Ongarora B.G., Fontenot K.R., Hu X., Sehgal I., Satyanarayana-Jois S.D., Vicente M.G.H. (2012). Phthalocyanine–Peptide Conjugates for Epidermal Growth Factor Receptor Targeting. J. Med. Chem..

[25] Mickler F.M., Möckl L., Ruthardt N., Ogris M., Wagner E., Bräuchle C. (2012). Tuning Nanoparticle Uptake: Live-Cell Imaging Reveals Two Distinct Endocytosis Mechanisms Mediated by Natural and Artificial EGFR Targeting Ligand. Nano Lett..

[26] Abourbeh G., Shir A., Mishani E., Ogris M., Rödl W., Wagner E., Levitzki A. (2012). PolyIC GE11 polyplex inhibits EGFR-overexpressing tumors. IUBMB Life.

[27] Agnes R.S., Broome A.M., Wang J., Verma A., Lavik K., Basilion J.P. (2012). An optical probe for noninvasive molecular imaging of orthotopic brain tumors overexpressing epidermal growth factor receptor. Mol. Cancer Ther..

[28] Chen C.J., Chan C.H., Lin K.L., Chen J.H., Tseng C.H., Wang P.Y., Chien C.Y., Yu H.M., Lin W.J. (2019). ^68^Ga-labelled NOTA-RGD-GE11 peptide for dual integrin and EGFR-targeted tumour imaging. Nucl. Med. Biol..

[29] Cheng L., Huang F.Z., Cheng L.F., Zhu Y.Q., Hu Q., Li L., Wei L., Chen D.W. (2014). GE11-modified liposomes for non-small cell lung cancer targeting: Preparation, ex vitro and in vivo evaluation. Int. J. Nanomed..

[30] Li X., Hu K., Liu W., Wei Y., Sha R., Long Y., Han Y., Sun P., Wu H., Li G. (2020). Synthesis and evaluation of [^18^F]FP-Lys-GE11 as a new radiolabeled peptide probe for epidermal growth factor receptor (EGFR) imaging. Nucl. Med. Biol..

[31] Rahmanian N., Hosseinimehr S.J., Khalaj A., Noaparast Z., Abedi S.M., Sabzevari O. (2017). ^99m^Tc-radiolabeled GE11-modified peptide for ovarian tumor targeting. DARU J. Pharma. Sci..

[32] Song S., Liu D., Peng J., Sun Y., Li Z., Gu J.-R., Xu Y. (2008). Peptide ligand-mediated liposome distribution and targeting to EGFR expressing tumor in vivo. Int. J. Pharmaceut..

[33] Grünwald G.K., Vetter A., Klutz K., Willhauck M.J., Schwenk N., Senekowitsch-Schmidtke R., Schwaiger M., Zach C., Wagner E., Göke B. (2013). EGFR-Targeted Adenovirus Dendrimer Coating for Improved Systemic Delivery of the Theranostic NIS Gene. Mol. Ther. Nucleic. Acids.

[34] Judmann B., Braun D., Schirrmacher R., Wängler B., Fricker G., Wängler C. (2022). Toward the Development of GE11-Based Radioligands for Imaging of Epidermal Growth Factor Receptor-Positive Tumors. ACS Omega.

[35] Judmann B., Wängler B., Schirrmacher R., Fricker G., Wängler C. (2023). Towards Radiolabeled EGFR-Specific Peptides: Alternatives to GE11. Pharmaceuticals.

[36] Striese F., Sihver W., Gao F., Bergmann R., Walther M., Pietzsch J., Steinbach J., Pietzsch H.-J. (2018). Exploring pitfalls of ^64^Cu-labeled EGFR-targeting peptide GE11 as a potential PET tracer. Amino Acids.

[37] Babich J.W., Fischman A.J. (1995). Effect of “co-ligand” on the biodistribution of 99mTc-labeled hydrazino nicotinic acid derivatized chemotactic peptides. Nucl. Med. Biol..

[38] Parry J.J., Kelly T.S., Andrews R., Rogers B.E. (2007). In vitro and in vivo evaluation of ^64^Cu-labeled DOTA-linker-bombesin(7–14) analogues containing different amino acid linker moieties. Bioconjug. Chem..

[39] Schlesinger J., Koezle I., Bergmann R., Tamburini S., Bolzati C., Tisato F., Noll B., Klussmann S., Vonhoff S., Wuest F. (2008). An 86Y-Labeled Mirror-Image Oligonucleotide: Influence of Y-DOTA Isomers on the Biodistribution in Rats. Bioconj. Chem..

[40] Schlesinger J., Rajander J., Ihalainen J.A., Ramesh D., Eklund P., Fagerholm V., Nuutila P., Solin O. (2011). Isomerism of [64Cu-NOTA-Bn]-Labeled Radiotracers: Separation of Two Complex Isomers and Determination of Their Interconversion Energy Barrier Using Ion Pair Chromatography. Inorg. Chem..

[41] García Garayoa E., Schweinsberg C., Maes V., Brans L., Bläuenstein P., Tourwé D.A., Schibli R., Schubiger P.A. (2008). Influence of the Molecular Charge on the Biodistribution of Bombesin Analogues Labeled with the [^99m^Tc(CO)_3_]-Core. Bioconj. Chem..

[42] Lee I., Kim M.H., Lee K., Oh K., Lim H., Ahn J.H., Lee Y.J., Cheon G.J., Chi D.Y., Lim S.M. (2023). Comparison of the Effects of DOTA and NOTA Chelators on ^64^Cu-Cudotadipep and ^64^Cu-Cunotadipep for Prostate Cancer. Diagnostics.

[43] Mitran B., Thisgaard H., Rinne S., Dam J., Azami F., Tolmachev V., Orlova A., Rosenström U. (2019). Selection of an optimal macrocyclic chelator improves the imaging of prostate cancer using cobalt-labeled GRPR antagonist RM26. Sci. Rep..

[44] Rinne S.S., Dahlsson Leitao C., Saleh-Nihad Z., Mitran B., Tolmachev V., Ståhl S., Löfblom J., Orlova A. (2020). Benefit of Later-Time-Point PET Imaging of HER3 Expression Using Optimized Radiocobalt-Labeled Affibody Molecules. Int. J. Mol. Sci..

[45] Hailing T., Yonghong P., Yufeng Z., Haitao T. (2022). Challenges for the application of EGFR-targeting peptide GE11 in tumor diagnosis and treatment. J. Control Release.

[46] Pethő L., Kasza G., Lajkó E., Láng O., Kőhidai L., Iván B., Mező G. (2020). Amphiphilic drug–peptide–polymer conjugates based on poly(ethylene glycol) and hyperbranched polyglycerol for epidermal growth factor receptor targeting: The effect of conjugate aggregation on in vitro activity. Soft Matter.

[47] Nuijens T., Piva E., Kruijtzer J.A.W., Rijkers D.T.S., Liskamp R.M.J., Quaedflieg P.J.L.M. (2012). Enzymatic C-terminal amidation of amino acids and peptides. Tetrahed. Lett..

[48] Wollack J.W., Zeliadt N.A., Mullen D.G., Amundson G., Geier S., Falkum S., Wattenberg E.V., Barany G., Distefano M.D. (2009). Multifunctional prenylated peptides for live cell analysis. J. Am. Chem. Soc..

[49] Serafini M., Pirali T., Tron G.C., Meanwell N.A., Lolli M.L. (2021). Chapter Three—Click 1,2,3-triazoles in drug discovery and development: From the flask to the clinic?. Advances in Heterocyclic Chemistry.

[50] Kovalová A., Pohl R., Vrabel M. (2018). Stepwise triple-click functionalization of synthetic peptides. Org. Biomol. Chem..

[51] Pearson D.A., Blanchette M., Baker M.L., Guindon C.A. (1989). Trialkylsilanes as scavengers for the trifluoroacetic acid deblocking of protecting groups in peptide synthesis. Tetrahed. Lett..

[52] Biet T., Avarvari N. (2014). Electroactive tetrathiafulvalene based pyridine-mono and -bis(1,2,3-triazoles) click ligands: Synthesis, crystal structures and coordination chemistry. CrystEngComm.

[53] Hosseinnejad T., Ebrahimpour-Malmir F., Fattahi B. (2018). Computational investigations of click-derived 1,2,3-triazoles as keystone ligands for complexation with transition metals: A review. RSC Adv..

[54] Mandel J.B., Douglas B.E. (1989). Crystal structure determination and strain analysis of cobalt(III) and copper(II)complexes of hexadentate and tetradentate ligands containing pyridyl arms. Inorg. Chim. Acta..

[55] Xie Q., Fu Q., Cui H.-B., Luo B.-H., Liu W.-Q., Qiao R., Ren Y.-J., Chen F., Zhang H.-X., Long J.-Q. (2020). Influence of the flexible tetrapyridines on electrocatalytic water oxidation by cobalt complexes. Polyhedron.

[56] Lin W., Fonseca Cabrera G.O., Aluicio-Sarduy E., Barnhart T.E., Mixdorf J.C., Li Z., Wu Z., Engle J.W. (2024). Radiolabeling Diaminosarcophagine with Cyclotron-Produced Cobalt-55 and [^55^Co]Co-NT-Sarcage as a Proof of Concept in a Murine Xenograft Model. Bioconj. Chem..

[57] Lin W., Śmiłowicz D., Joaqui-Joaqui M.A., Bera A., Zhong Z., Aluicio-Sarduy E., Mixdorf J.C., Barnhart T.E., Engle J.W., Boros E. (2024). Controlling the Redox Chemistry of Cobalt Radiopharmaceuticals. Angew. Chem. In. Ed..

[58] Vad M.S., Nielsen A., Lennartson A., Bond A.D., McGrady J.E., McKenzie C.J. (2011). Switching on oxygen activation by cobalt complexes of pentadentate ligands. Dalton Trans..

[59] Ségaud N., Rebilly J.-N., Sénéchal-David K., Guillot R., Billon L., Baltaze J.-P., Farjon J., Reinaud O., Banse F. (2013). Iron Coordination Chemistry with New Ligands Containing Triazole and Pyridine Moieties. Comparison of the Coordination Ability of the N-Donors. Inorg. Chem..

