# Supplementary material for: Radiocobalt-Labeling of a Polypyridylamine Chelate Conjugated to GE11 for EGFR-Targeted Theranostics
Source: Molecules. 2025 Jan 7;30(2):212. doi: 10.3390/molecules30020212 (PMC11767697; doi:10.3390/molecules30020212)
Supplement: Supplementary file 1 [file molecules-30-00212-s001.zip › molecules-3386921-supplementary.pdf]

Supplementary Information

# Radiocobalt-Labeling of a Polypyridylamine Chelate Conjugated to GE11 for EGFR-Targeted Theranostics

**Lorraine Gaenaelle Gé [1,2,†], Mathias Bogetoft Danielsen [3,†], Aaraby Yoheswaran Nielsen [1], Mathias Lander Skavenborg [3], Niels Langkjær [1], Helge Thisgaard [1,2,*] and Christine J. McKenzie [3,*]**

[1]Department of Nuclear Medicine, Odense University Hospital, Kloevervaenget 47, 5000 Odense C, Denmark; lorrainege94@hotmail.com (L.G.G.); aaraby.yoheswaran.nielsen@rsyd.dk (A.Y.N.); niels.langkjaer@rsyd.dk (N.L.)

[2]Department of Clinical Research, University of Southern Denmark, Campusvej 55, 5230 Odense M, Denmark

[3]Department of Physics, Chemistry and Pharmacy, University of Southern Denmark, Campusvej 55, 5230 Odense M, Denmark; mbd@sdu.dk (M.B.D.); skavenborg@sdu.dk (M.L.S.)

*Correspondence: helge.thisgaard@rsyd.dk (H.T.); mckenzie@sdu.dk (C.J.M.)

[†]These authors contributed equally to this work.

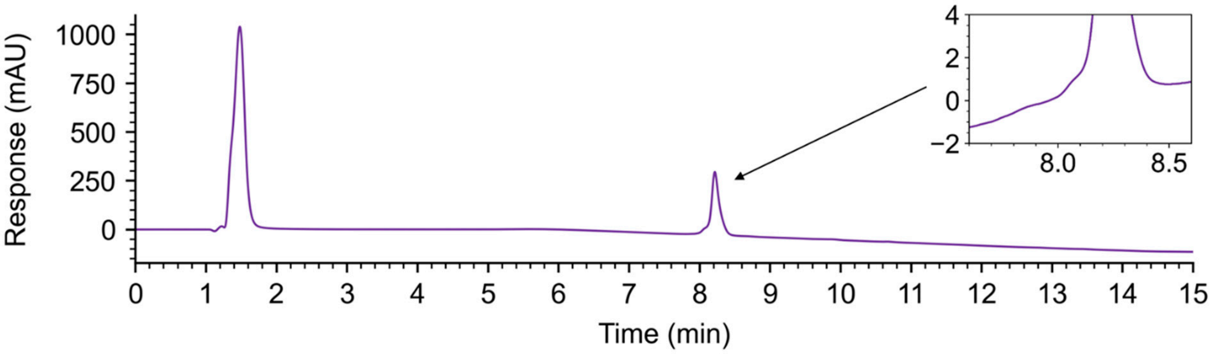


**Figure S1**: UV chromatogram following analytical RP-HPLC of TZTPEN-GE11 dissolved in DMSO and diluted in MQ water (1:19). Two peaks are observed: one at 1.48 min and one at 8.22 min with a small shoulder present at approximately 0.15 min before the main peak.

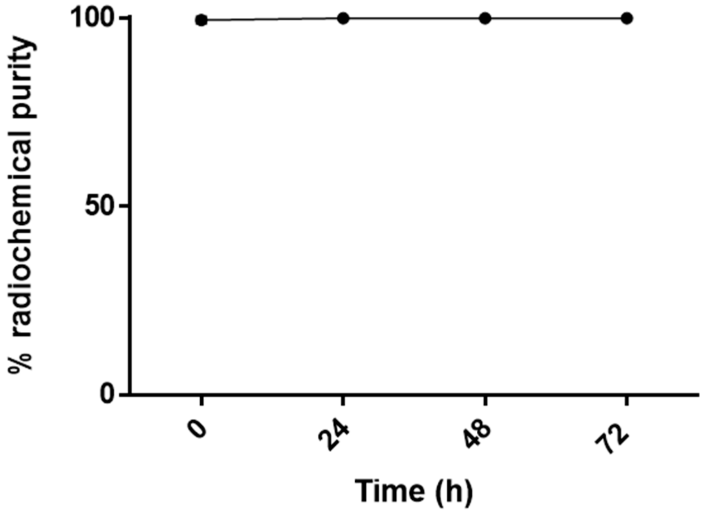


**Figure S2:** Stability of [$^{57}$Co]Co-TPEN-GE11 (1.13-1.38 MBq/nmol, RCY > 98.92%) in PBS for up to 72 h, at pH 5. The radiochemical purity was determined by RP-HPLC analysis. Data is presented as Mean ± SD, *n* = 3.
